# Screening of hub inflammatory bowel disease biomarkers and identification of immune-related functions based on basement membrane genes

**DOI:** 10.1186/s40001-023-01193-5

**Published:** 2023-07-22

**Authors:** Penghang Lin, Jin Hua, Zuhong Teng, Chunlin Lin, Songyi Liu, Ruofan He, Hui Chen, Hengxin Yao, Jianxin Ye, Guangwei Zhu

**Affiliations:** 1 Department of Gastrointestinal Surgery 2 Section, Institute of Abdominal Surgery, Key Laboratory of Accurate Diagnosis and Treatment of Cancer, The First Affiliated Hospital of Fujian Medical University, National Regional Medical Center, Binhai Campus of the First Affiliated Hospital, Fujian Medical University, 20th, Chazhong Road, Fuzhou, 350005 Fujian China; 2 Key Laboratory of Ministry of Education for Gastrointestinal Cancer, Fujian Medical University, Fuzhou, 350000 China

**Keywords:** Basement membrane, IBD, Biomarkers, CD, UC, SVM-RFE

## Abstract

**Background:**

Inflammatory bowel disease (IBD), including Crohn's disease (CD) and ulcerative colitis (UC), is a chronic, inflammatory, and autoimmune disease, but its specific etiology and pathogenesis are still unclear. This study aimed to better discover the causative basement membrane (BM) genes of their subtypes and their associations.

**Methods:**

The differential expression of BM genes between CD and UC was analyzed and validated by downloading relevant datasets from the GEO database. We divided the samples into 3 groups for comparative analysis. Construction of PPI networks, enrichment of differential gene functions, screening of Lasso regression models, validation of ROC curves, nomogram for disease prediction and other analytical methods were used. The immune cell infiltration was further explored by ssGSEA analysis, the immune correlates of hub BM genes were found, and finally, the hub central genes were screened by machine learning.

**Results:**

We obtained 6 candidate hub BM genes related to cellular immune infiltration in the CD and UC groups, respectively, and further screened the central hub genes ADAMTS17 and ADAMTS9 through machine learning. And in the ROC curve models, AUC > 0.7, indicating that this characteristic gene has a more accurate predictive effect on IBD. We also found that the pathogenicity-related BM genes of the CD and UC groups were mainly concentrated in the ADAMTS family (ADAMTS17 and ADAMTS9). Addition there are some differences between the two subtypes, and the central different hub BM genes are SPARC, POSTN, and ADAMTS2.

**Conclusions:**

In the current study, we provided a nomogram model of CD and UC composed of BM genes, identified central hub genes, and clarified the similarities and differences between CD and UC. This will have potential value for preclinical, clinical, and translational guidance and differential research in IBD.

**Supplementary Information:**

The online version contains supplementary material available at 10.1186/s40001-023-01193-5.

## Introduction

Inflammatory bowel disease (IBD) is an inflammatory disease involving the ileum, rectum, and colon, among which ulcerative colitis (UC) and Crohn's disease (CD) are the most common. As a systemic disease, IBD affects the quality of life of patients, and severe cases increase the risk of colon cancer [1, 2]. The main pathological feature of IBD is intestinal inflammation and causes varying degrees of intestinal mucosal damage, including extensive epithelial cell death, ulcers, crypt abscesses, and the formation of fibrosis [3, 4]. At present, the pathogenesis of IBD is still unclear, and the possible causes include genetic susceptibility, commensal flora disturbance, epithelial barrier defect, immune dysregulated response, and environmental factors [5]. Therefore, it is of great significance to further explore new molecular markers and molecular mechanisms of IBD.

The BM is the evolutionary conserved animal extracellular matrix and forms sheet-like structures that epithelialize and surround most tissues [6–8]. Two independent planar networks of laminin and type IV collagen molecules associate with cell surface interactors and provide a scaffolding structure for building BMs along tissues. These functions will suggest that the BM has an important relationship with the occurrence and development of IBD [9]. Recently, Jayadev et al.[10] systematically defined 222 humans BM genes, which identified the enormous complexity of BM and important impact on human health. We will further use these 222 humans BM genes to explore new molecular markers of BM closely related to IBD. BM has a variety of components that can be used to resist mechanical stress, determine tissue shape, and create diffusion barriers. They also provide cues directing cell polarity, differentiation, migration, and survival. Variations in more than 20 BM genes underline their diverse and fundamental functions that underlie human disease. BM proteins are targets of autoantibodies in immune diseases, and defects in BM protein expression and turnover are hub pathogenic aspects of cancer, inflammation, and fibrosis [11].

Advances in high-throughput sequencing technologies such as RNA-seq and microarrays have provided opportunities to comprehensively characterize the molecular features of tumorigenesis [12]. Furthermore, the application of these high-throughput technologies has facilitated the identification of promising biomarkers for cancer diagnosis and prognostic assessment. Appropriate IBD data were obtained through Gene Expression Omnibus (GEO). Based on BM genes, we grouped and compared them separately, performed a series of bioinformatics analyses, and mined new molecular markers to predict related drugs and miRNA, which aims to lay the foundation for the development of IBD treatment, identification of subtypes, and further in-depth research.

## Materials and methods

### Data sources and processing methods

IBD samples with complete gene expression information were downloaded from GEO (https://www.ncbi.nlm.nih.gov/). The dataset GSE165512 (Public on Nov, 2021 GPL16791) used for the analysis collected 46 normal patient samples, 84 Crohn's patient samples, and 40 ulcerative colitis patients. All tissue samples were obtained from the patient's ileocolic tissue. Microarray data were downloaded and reanalyzed from the public GEO database, and systematic bioinformatics analysis was performed. Dataset GSE179285 (Public on Jul, 2021 GPL6480) was used for validation of the analysis. The numerically larger expression values of the dataset were log2 transformed. In this study, no experiments were performed on humans or animals. Therefore, ethical approval or consent to participate was not applicable.

### Analysis of differential expression BM genes

The expression datasets were extracted by R software for BM genes, and the "limma" package was applied to select differential expression genes (DEGs) between Crohn's disease or UC and control samples, respectively [13]. The "limma" package is a mainstream tool for genetic differential analysis [14]. We set significance criteria as |logFC| > 0.5 and *p* < 0.05 and visualized DEGs via heatmaps and volcano plots.

### PPI network analysis

As described above, we selected significantly differential expression BM genes from CD or UC. We mapped a protein–protein interaction (PPI) network using the online mapping tool "STRING" (https://string-db.org/cgi/input.pl) with a minimum interaction distance of 0.9. Next, Cytoscape (v3.9.1) visualized the network model and used CytoHubba in the app to calculate the 10 top of hub genes.

### Functional analysis of DEG

To reveal the biological functions of the selected DEGs, Gene Ontology (GO) enrichment analysis including biological process (BP), cellular component (CC), and molecular function (MF) analysis was performed using the "clusterProfiler" package. Criterion was set at *p* < 0.05 and results were visualized in bubble charts. In addition, "GObubble" and "GOChord" were applied with the "GOplot" package to illustrate functional analysis data. Likewise, the Kyoto Encyclopedia of Genes and Genomes (KEGG) is a database that integrates genomic, chemical and phylogenetic functional information [15], which analysis illustrates the enrichment of pathways in DEGs by applying the 'clusterProfiler' package; results are presented as bar and circle graphs. To further study the differential expression immune environment of the disease, GSEA used the immune-related gene set in the Molecular Signatures Database (MsigDB) as a reference, in which the gene set with *p* < 0.05 and false discovery rate (FDR) *q* value < 0.05 was considered to be significantly enriched. GSEA enrichment analysis was performed by the "limma" and "enrichplot" packages and the top 5 immune gene sets are displayed [16].

### Screening and identification of Lasso prediction models

Lasso logistic regression is a machine learning method that identifies variables by finding the λ value with the smallest classification error. The partial-likelihood bias of log-change plotted by Lasso regression in tenfold cross-validation. A dashed vertical line is drawn at the optimal value with the minimum criterion (lambda. min) and 1 standard error of the minimum criterion (1-se criterion). The "glmnet" package is a package used to construct generalized linear and similar models, and candidate hub genes were then crossed with DEGs using the "glmnet" package of the R software for Lasso analysis to screen the final hub genes [17]. The hub gene expression levels in the BM of IBD patients were assessed using boxplots. The ROCs of the screened hub genes were drawn and their AUCs were determined.

### Drawing of a nomogram

Nomograms were constructed and displayed using the "regplot" R package. Then, calibration plots were drawn to assess the reliability of the nomogram, and decision curve analysis (DCA) was performed to investigate the net clinical benefit of the nomogram (R packages "caret" and "rmda"). The occurrence and development of IBD are evaluated by hub genes.

### Analysis of immune cells and functions infiltration

The degree of immune cell and functions infiltration can be quantified by ssGSEA analysis [18]. The relative infiltration levels of immune cells and functions in the GSE165512 dataset were quantified using the ssGSEA algorithm, and heatmaps and boxplots were drawn showing the correlation and differential expression levels of immune infiltrating cells and functions. The relationship between hub genes and immune cells and functions was analyzed with Spearman correlation of immune infiltrating cells with hub genes and then visualized using the "ggplot2" software package.

### Predict related drugs and miRNAs

We screened out the hub BM-related genes to IBD through the online Enrichr database (https://maayanlab.cloud/Enrichr/) to predict related drugs and miRNAs, and screened the top 10 drugs or miRNAs with *p* < 0.05 for visualization. The miRNA network relationship was constructed by Cytoscape (v3.9.1) to build a visual network model.

### Screening of machine learning models

Support vector machines-recursive feature elimination (SVM-RFE) is to train the sample by the model, then rank each feature with a score, remove the feature with the smallest feature score, then train the model again with the remaining features for the next iteration, and finally select the required number of features, which is often used for the extraction of disease feature markers [19]. We selected differential expression BM genes from the modules most closely related to CD or UC for machine learning. We run the e1071 package to eliminate recursive features of the obtained differential genes and use the SVM-RFE function for data calculations. We set up folds by wrapping the entire feature selection and generalization error estimation process in a top loop of outer cross-validation. Finally, we used the loop function to estimate the generalization error of different numbers of top features with a 10 × CV standard, so that the error rate reaches the lowest point, and the best gene features are obtained.

## Result

### Study design and data preliminary analysis

**Fig. 1:**
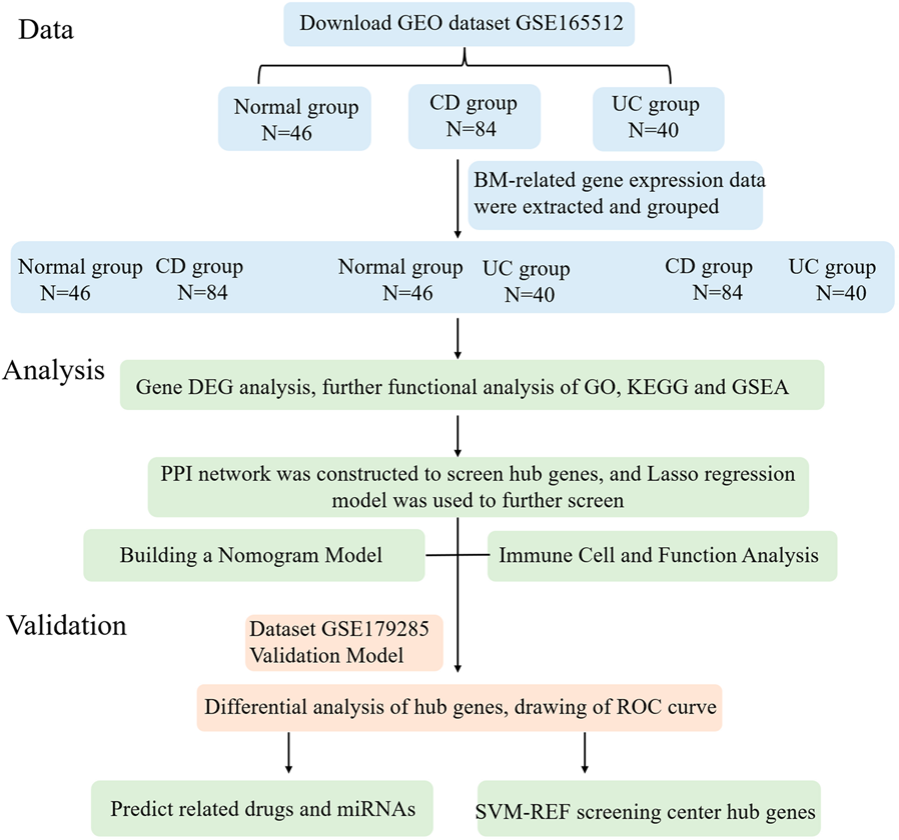
Workflow of data processing

The overall design idea of this study is shown in the flowchart (Fig. 1). The samples of dataset GSE165512 were divided into normal group (*n* = 46), CD group (*n* = 84), and UC group (*n* = 40), which were used for subsequent model construction and analysis, respectively. To investigate the relationship between inflammatory bowel disease and basement membrane, we first extracted the 222 BM gene expression data found by Jayadev et al.[10]. Probably due to the tissue specificity of BM genes a few that were undetected with very low gene expression in the intestine were excluded. Of these, 211 genes were extracted in the Con and CD groups; 221 genes in the Con and UC groups; and 211 genes in the CD and UC groups. The expression matrix of the extracted BM genes was used for further analysis.

### Differential expression of BM genes and screening of hub genes

**Fig. 2:**
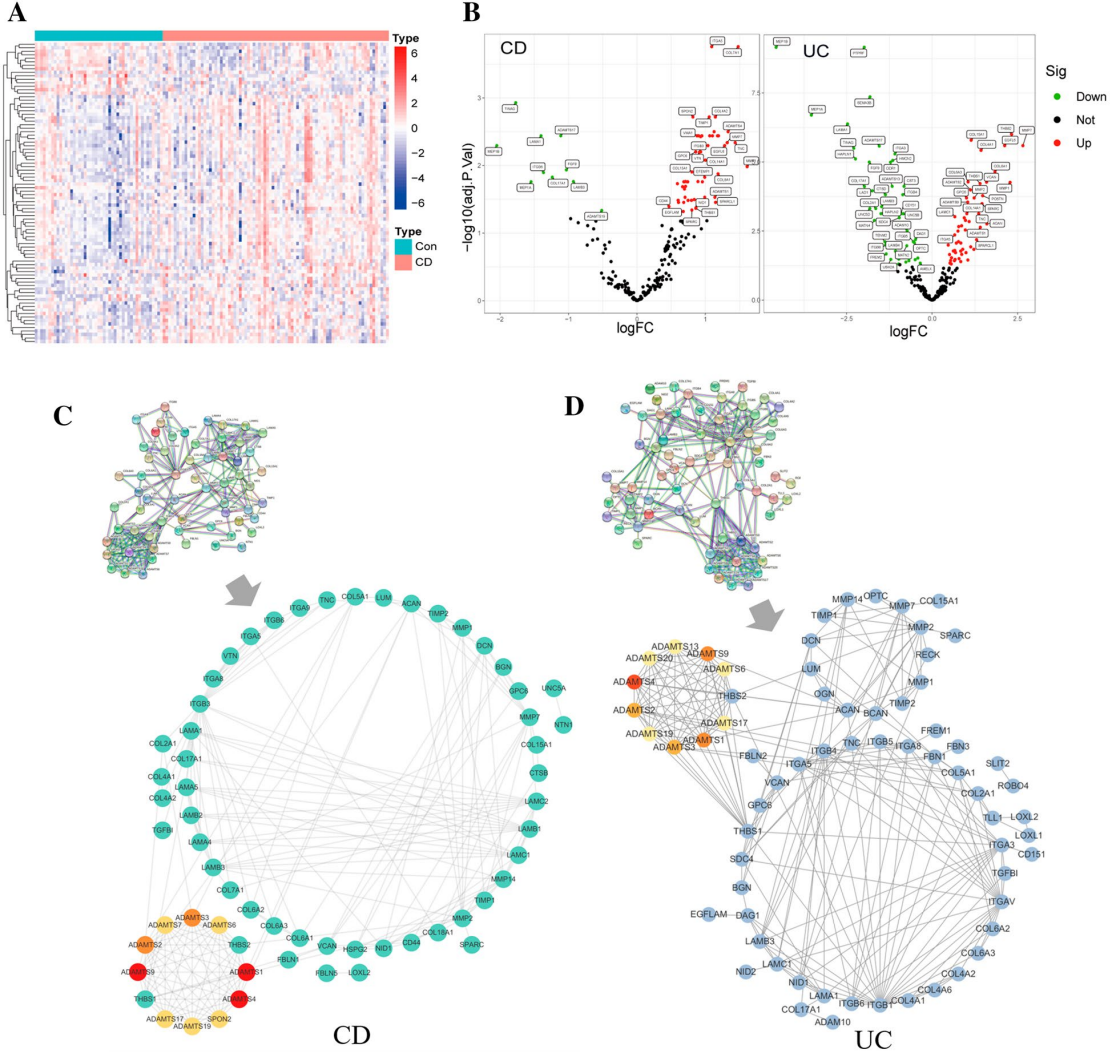
Data variance analysis and PPI network construction. **A** Heatmap of differential expression of 211 BM genes extracted from the CD group. **B** Volcano plot of BM genes in CD and UC groups. **C**, **D** PPI network construction of BM differential genes in CD and UC groups

The differential expression of BM genes between normal and CD groups, normal and UC, and CD and UC groups were analyzed by the “limma” package in R language (|logFC| > 0.50, *p* < 0.05), and a heat map and a volcano plot were drawn (Fig. 2A, B and Additional file 1: Fig. S1A, B). 83, 101, and 56 significantly differential expressed BM genes were found in these three groups, respectively (Additional file 2: Tables S2, S4, S6). String is an online website tool for analyzing protein–protein interactions (PPI) (https://cn.string-db.org/) [20]. The differential genes were passed through the String online database, and the standard set was that the interaction score of the network was > 0.9 to construct PPI network diagrams (Fig. 2C, D and Additional file 1: Fig. S1C), and calculated by the CytoHubba(v0.1) plugin in Cytoscape, and obtained the hub top 10 genes respectively. Red indicates hub genes, and darker colors indicate higher scores (Table 1).

**Table 1:** BM hub genes in IBD

	CD group		UC group		CD/UC group	
Rank	CytoHubba	Lasso	CytoHubba	Lasso	CytoHubba	Lasso
1	ADAMTS4	ADAMTS4	ADAMTS4	ADAMTS4	LUM	LUM
2	ADAMTS9	–	ADAMTS9	ADAMTS9	POSTN	POSTN
3	ADAMTS1	–	ADAMTS1	–	MMP2	–
4	ADAMTS2	–	ADAMTS2	ADAMTS2	COL5A1	COL5A1
5	ADAMTS3	ADAMTS3	ADAMTS3	ADAMTS3	VCAN	VCAN
6	ADAMTS17	ADAMTS17	ADAMTS17	ADAMTS17	FBLN2	–
7	ADAMTS19	ADAMTS19	ADAMTS19	–	SPARC	SPARC
8	SPON2	SPON2	ADAMTS20	–	CD44	–
9	ADAMTS6	ADAMTS6	ADAMTS6	–	LOXL1	LOXL1
10	ADAMTS7	–	ADAMTS13	ADAMTS13	ADAMTS2	ADAMTS2

### Lasso regression model to screen the central hub genes

**Fig. 3:**
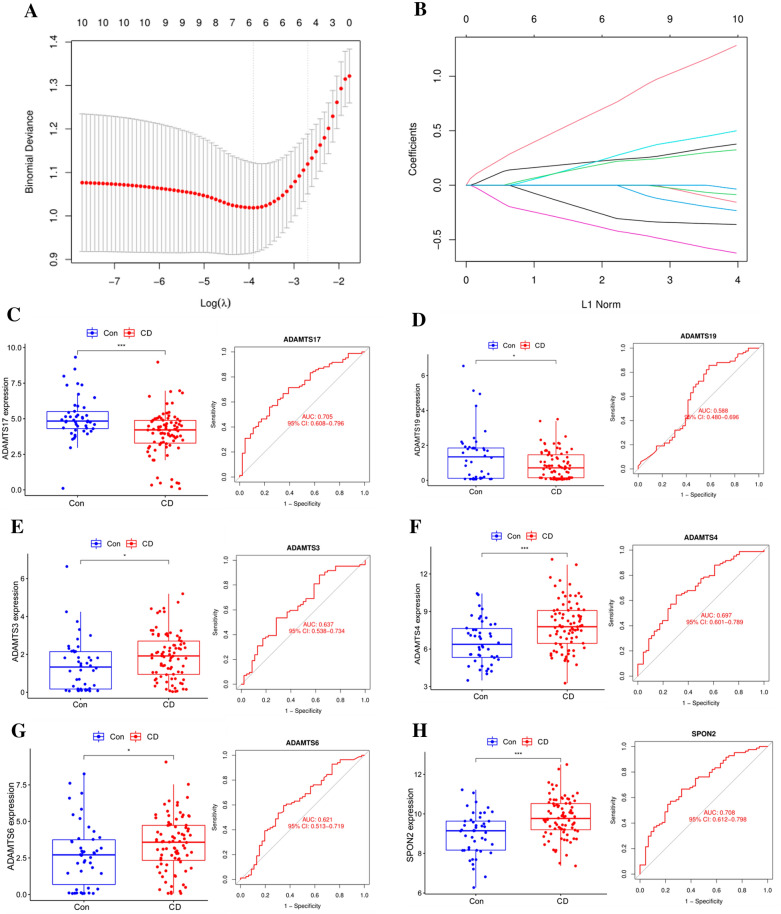
DEGs identification and hub gene screening. **A** Lasso coefficient profile of tenfold cross-validation hub genes in the CD group. **B** Partial likelihood bias of log-change plotted by Lasso regression in tenfold cross-validation. A dashed vertical line is drawn at the optimal value with the minimum criterion (lambda. min) and 1 standard error of the minimum criterion (1-se criterion). **C**–**H** Boxplots of 6 hub genes in CD group and construction of ROC curve model (*p < 0.05; *p < 0.05; *p < 0.05, with an unpaired Student’s *t* test)

Lasso regression is commonly used for screening diagnostic model genes or prognostic model genes and is based on general linear regression with a canonical term added to ensure best-fit error while making the model generalizable [21]. By building a Lasso model in the training set, the value of the hyperparameter λ is obtained by tenfold cross-validation using the smallest criterion. After adjusting the cross-validation of parameter selection in Lasso regression, the final hub genes were obtained, which were ADAMTS4, ADAMTS3, ADAMTS17, ADAMTS19, SPON2, and ADAMTS6 in the CD group, respectively. Similarly, the final hub genes of the UC group and the CD/UC group can be obtained (Fig. 3A, B and Additional file 1: Fig. S1D–F) (Table 1).

The expression levels of hub genes in each group were verified by boxplots, and the expression differences of the central genes were visually displayed, and the expression of these genes in tissues was compared with the control group, and the results were all statistically significant (*p* < 0.05). The Receiver operating characteristic curve (ROC) is used to evaluate the effectiveness of a marker in classifying or diagnosing two types of test subjects (for instance patients and normal individuals). The area under the curve (AUC) is calculated (0.5 = purely random prediction and 1 = full discrimination) to find the optimal cut-off value for this model [22]. ADAMTS4, ADAMTS3, ADAMTS17, ADAMTS19, SPON2, and ADAMTS6 were then validated with ROC curves and AUC statistics to assess the ability to differentiate from healthy controls and found to have good sensitivity and specificity. The National Institutes of Health define an AUC > 0.6 as "FAIR"[23, 24]. The horizontal coordinates represent specificity and the vertical coordinates represent sensitivity, where the AUC of SPON2 was 0.71, cut-off value was 0.35, specificity (67.11%) and sensitivity (67.90%), suggesting that this gene has a good predictive effect and better diagnostic ability for CD (Fig. 3C–H).

**Fig. 4:**
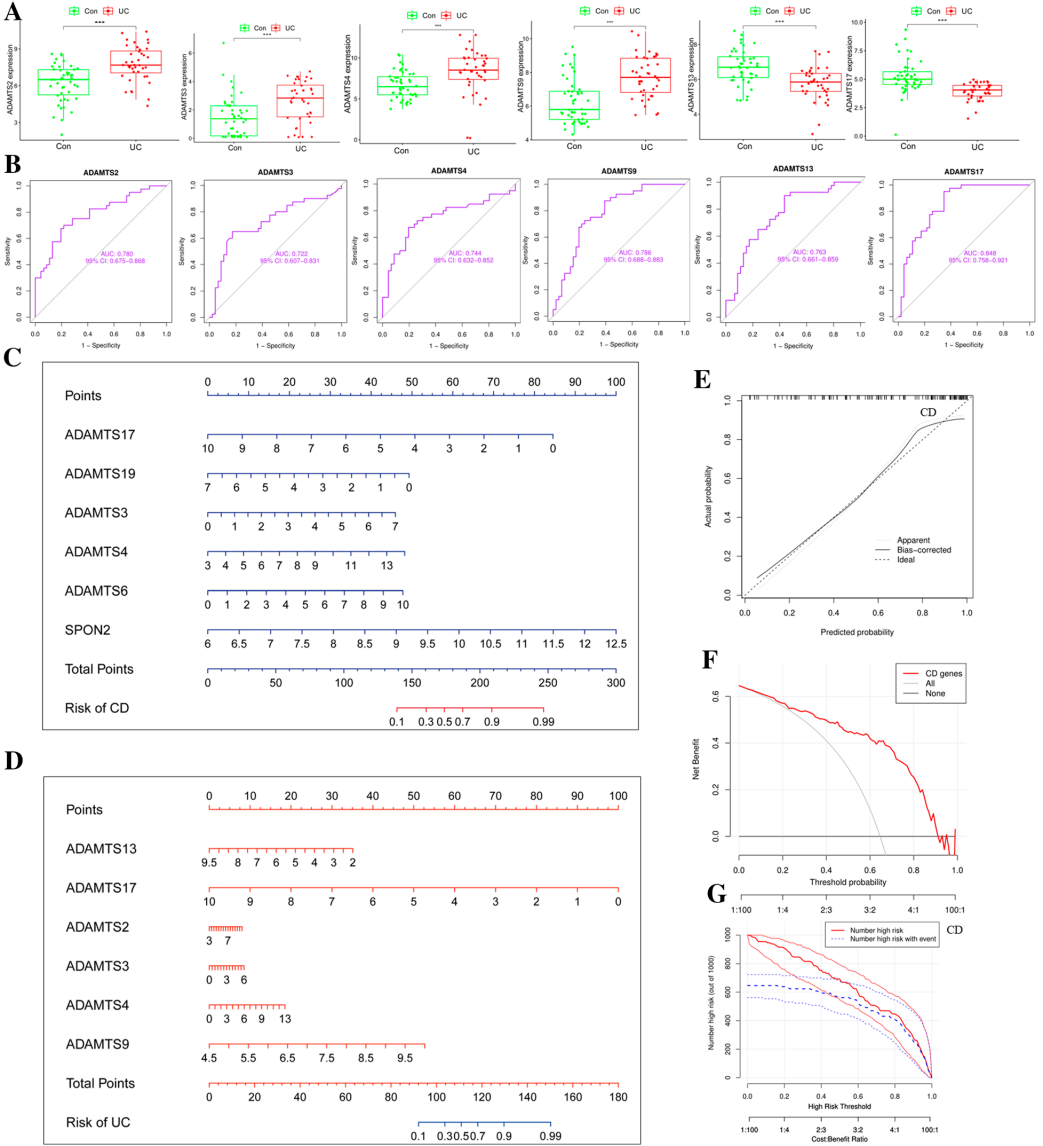
Drawing of hub gene nomograms. **A** Boxplots of hub genes in the UC group. **B** Drawing of the ROC model of hub genes in the UC group. **C** Plotting of the nomogram of hub genes in the CD group. **D** Drawing of the nomogram of hub genes in the UC group. **E**–**G** Prediction curve, decision curve, and clinical impact curve of the nomogram model in the CD group (*p < 0.05; *p < 0.05; *p < 0.05, with an unpaired Student’s *t* test)

In the UC group, the hub genes ADAMTS4, ADAMTS9, ADAMTS2, ADAMTS3, ADAMTS17, and ADAMTS13 were screened by Lasso regression for boxplots verification, all the results were significantly different and the ROC and AUC statistics were verified. Among these, the AUC of ADAMTS17 was 0.85, with a cut-off value of 0.60, specificity (94.80%) and sensitivity (64.60%), suggesting a good predictive effect (Fig. 4A, B). Similarly, it can be verified in the CD/UC group. The expression of SPARC gene was significantly higher in the UC group than in the CD group, with an AUC value of 0.71, cut-off value of 0.40, specificity (70.35%) and sensitivity (69.70%). It is indicated that SPARC gene is important for the identification of two IBD subtypes (Additional file 1: Fig. S2).

### Construction of nomogram to predict disease

The nomogram model was constructed to make more accurate personalized predictions for patients. We chose to establish a nomogram model of CD with BM hub genes (including ADAMTS4, ADAMTS3, ADAMTS17, ADAMTS19, SPON2, and ADAMTS6) (Fig. 4C), in which the predicted curve almost coincides with the actual curve, indicating a good prediction effect (Fig. 4E). Both the DCA decision curve and the clinical impact curve showed that the model had good accuracy and could accurately predict the occurrence and development of CD (Fig. 4F, G) [25]. Also available, in the UC group, BM hub genes (including ADAMTS4, ADAMTS9, ADAMTS2, ADAMTS3, ADAMTS17, and ADAMTS13) were constructed for nomograms (Fig. 4D and Additional file 1: Fig. S3A–C).

### Functional enrichment analysis of DEGs

**Fig. 5:**
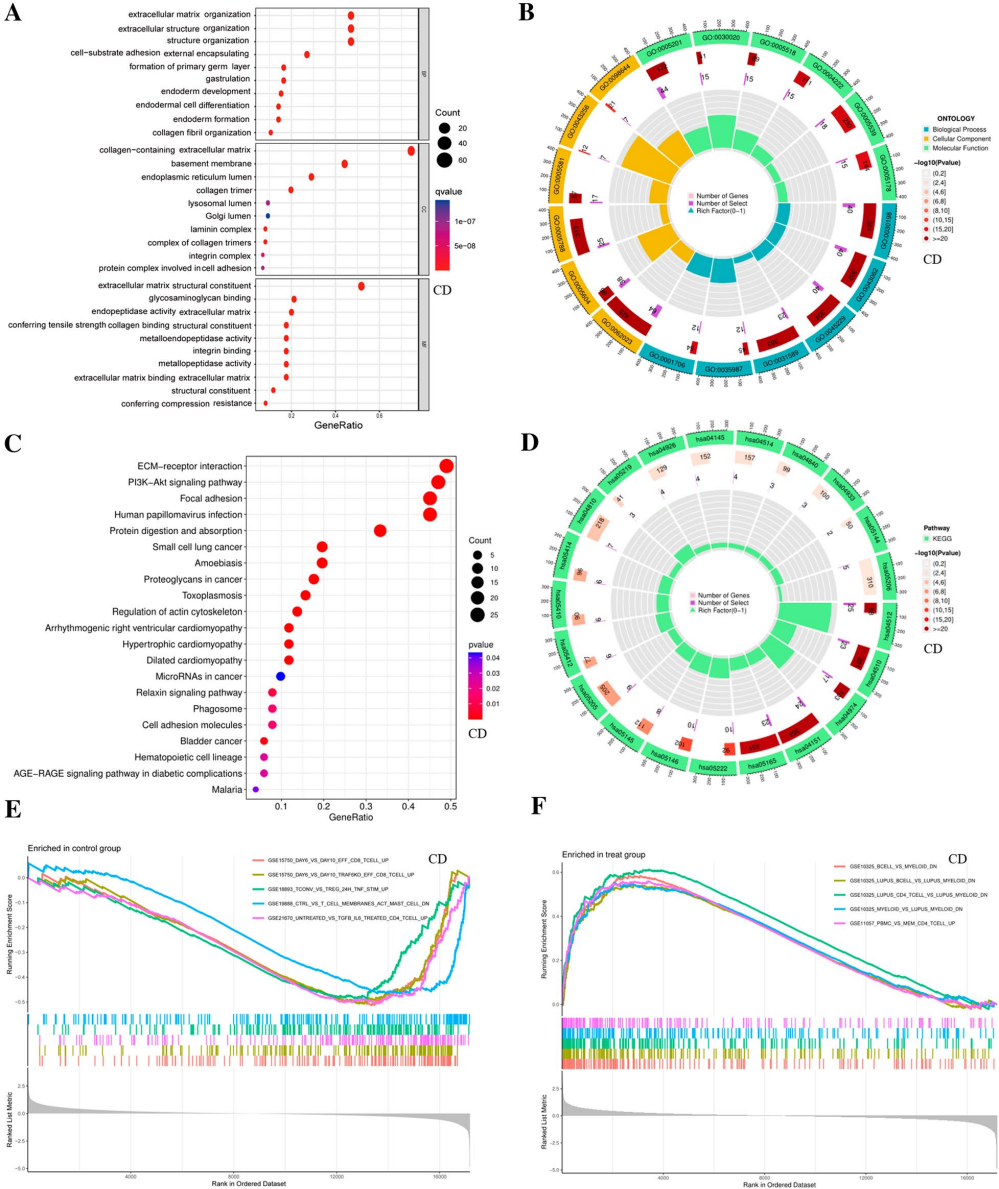
Functional analysis of DEGs. **A** Bubble chart of GO enrichment analysis of differential BM genes in the CD group, and the top 10 functions are listed respectively. **B** Circle chart of GO enrichment analysis of differential BM genes in CD group and the distribution of top 6 functions are listed respectively. **C** Bubble chart of differential BM genes KEGG enrichment analysis in CD group and the top 10 functional pathways are listed. **D** Circle plot of CD group differential BM gene KEGG enrichment analysis. **E**, **F** In the CD group analysis, GSEA analysis was performed with the immune-related gene set as a reference, and the top 5 enriched immune gene sets were listed, respectively

Through GO and KEGG analysis, the biological function, cellular localization, and molecular function of DEGs of BM genes in CD patients were found. It can be concluded that the biological functions of the differential expression genes are mainly concentrated in the extracellular matrix organization, structural organization, and collagen fiber organization; The cell localization is mainly concentrated in collagen extracellular matrix; Molecular functions are mainly focused on antitension and strength of extracellular matrix structural components and collagen binding (Fig. 5A, B). Analysis of the KEGG signaling pathway revealed that the functional pathways of differential expression genes were mainly focused on ECM-receptor interactions and focal adhesions to perform biological functions (Fig. 5C, D). Taken together, these results identify biological processes and signaling pathways associated with significant differential BM in CD patients. The Molecular Signatures Database (MsigDB) is a database of annotated gene sets of all types and can be used with GSEA software. GSEA uses the “immunesigdb.gmt” dataset in MsigDB as a reference [26]. It is a dataset with expression levels of various gene pairs in immune cells. We can obtain that the main enriched immune cell infiltration is negatively correlated with the normal group and positively correlated with the CD group. It further suggests that the development of CD is associated with BM genes regulating immune cell levels and causing abnormal aggregation of immune cells in CD intestinal tissues, implying that BM genes regulating immune cells are closely associated with the development of the disease (Fig. 5E, F).

In the UC group, the biological functions of genes differential expression in BM were also mainly concentrated in an extracellular matrix organization, structural organization, and collagen fiber organization. The localization of cells is mainly concentrated in collagen extracellular matrix. Molecular functions mainly focus on metalloendopeptidase activity and glycosaminoglycan (Additional file 1: Fig. S3D).

Analysis of the KEGG signaling pathway revealed that the functional pathways of differential expression genes were mainly focused on ECM-receptor interaction and biological functions on focal adhesions (Additional file 1: Fig. S3E). It is interesting to find that in the bioinformatics analysis of base BM genes, the enrichment results of CD and UC subtypes have highly similar biological functions. Also in the GSEA, we found differentially enriched results of immune cell infiltration (Additional file 1: Fig. S3F, G). In the CD/UC group, we found similarities in biological function enrichment. It can be explained that the functional changes caused by the two subtypes are similar, but the degree is inconsistent, and there is a certain enrichment difference, which may be explained by the specificity of their respective diseases (Additional file 2: Table S7).

### Immune cell infiltration and its correlation with the hub gene

**Fig. 6:**
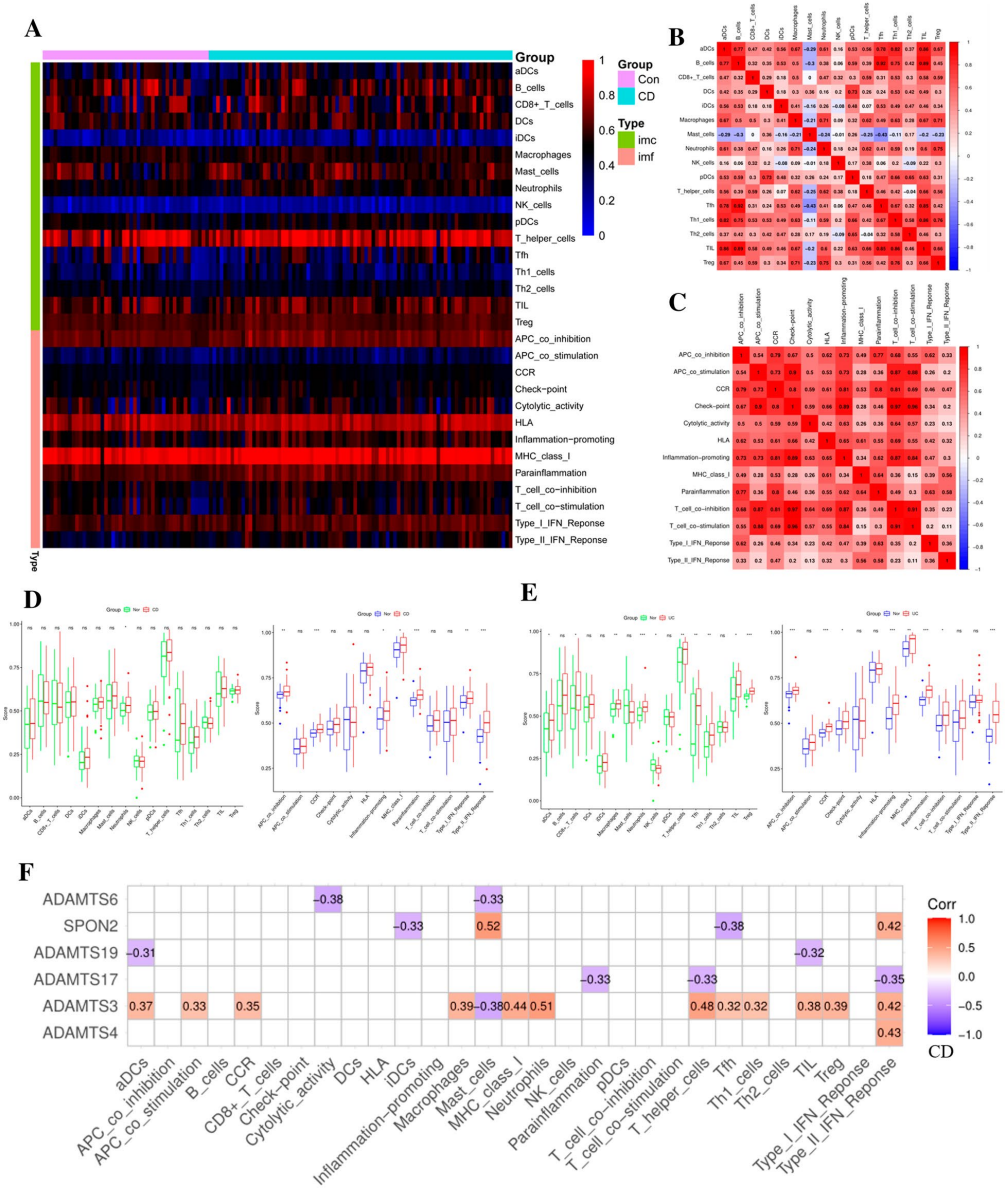
Immune cells and functions correlation analysis. **A** In the CD group, the heatmap shows the distribution of immune cells and functions within the group by ssGSEA analysis. **B**, **C** Correlation between immune cells and functions within the group was shown by ssGSEA analysis. **D**, **E** The levels of immune cells and functions within each group were shown by ssGSEA analysis in the CD and UC groups, respectively. **F** The relationship between six central hub BM genes and immune cells and functional infiltration in the CD group by ssGSEA analysis (*p < 0.05; *p < 0.05; *p < 0.05, with an unpaired Student’s *t* test)

Single sample GSEA analysis(ssGSEA) quantified immune infiltration from different angles, is a special type of GSEA analysis [27]. To further investigate the differences in BM immune cell infiltration among CD patients, the ssGSEA algorithm was used to evaluate the relationship between the two, and heatmap and correlation map of immune cells and functions infiltration were drawn. It can be found that most immune cells and functions were positively correlated, and the correlation coefficient is > 0.80 (Fig. 6A–C). Next, the distribution of immune cells and function is shown. In CD, neutrophils were significantly higher than normal (*p* < 0.05), and there were more changes in immune functions (Fig. 6D).Also in the UC group and the CD/UC group, we performed analyses (Fig. 6E and Additional file 1: Fig. S4A–D). Six hub BM genes were tested for the correlation between immune cells and functions, among which ADAMTS3 was associated with a variety of immune cells and functions (Fig. 6F). We performed the same analysis in the UC group and the CD/UC group (Additional file 1: Fig. S4E). These results provide further evidence that these immune cells play a crucial role in the progression of IBD.

### Validation of hub immune-related BM genes in CD and UC

**Fig. 7:**
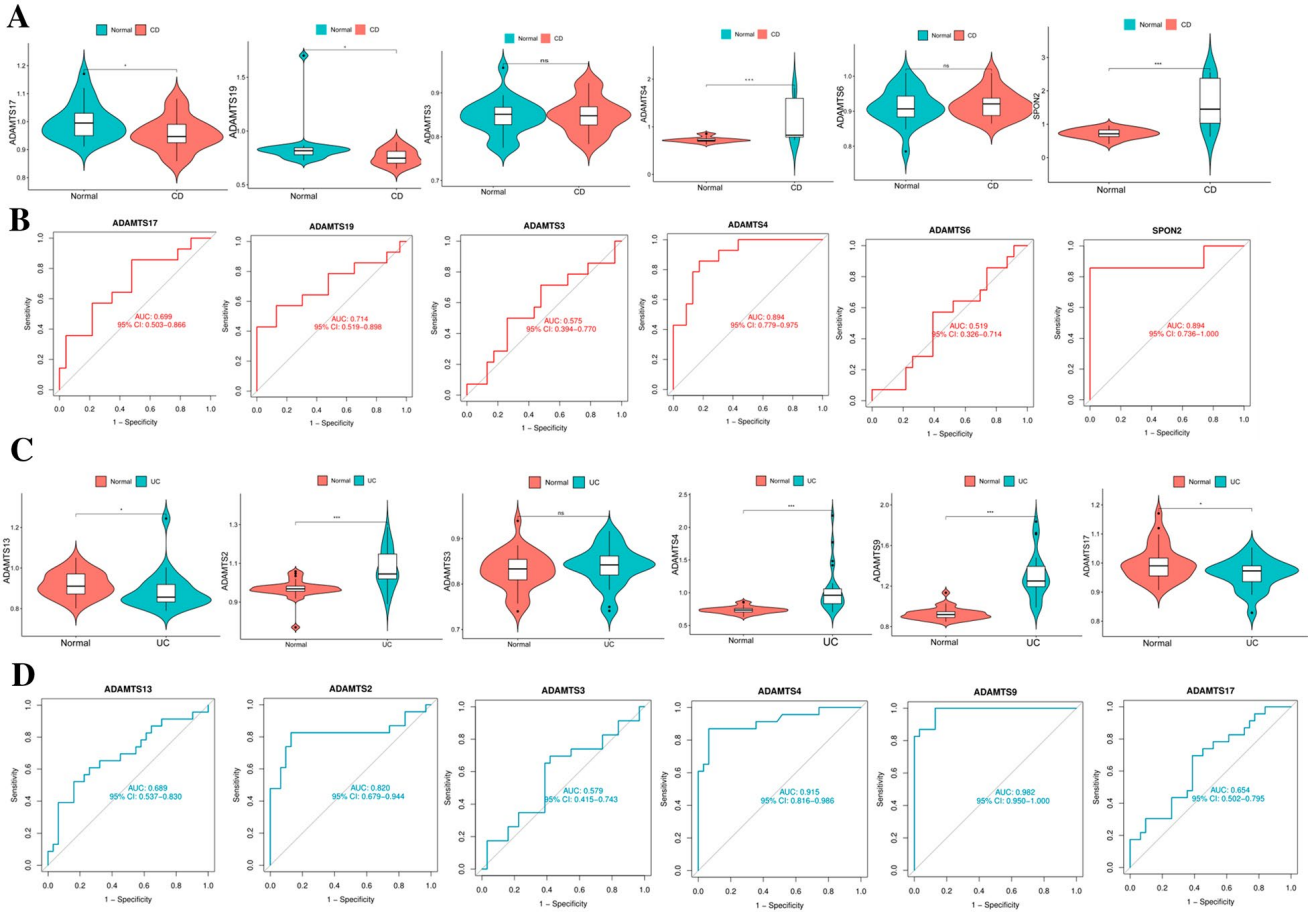
Validation of hub BM genes. **A**, **B** In the CD group, boxplots and ROCs verify the expression of hub BM genes. **C**, **D** In the UC group, boxplots and ROCs verify the expression of hub BM genes (*p < 0.05; *p < 0.05; *p < 0.05, with an unpaired Student’s *t* test)

The IBD dataset GSE179285 of the GEO database was used to validate the model screening results. The expression levels of the central genes in each group were verified by box plots. We can see that the expression differences of all predicted genes are consistent with the validation group, indicating the accuracy and validity of the model predictions. However, ADAMTS3 and ADAMTS6 in the CD group and ADAMTS3 in the UC group were not statistically significant. There were a few discrepancies in the results of the dataset validation, which may have been caused by the variability of batches and individuals from different sample sources is a slight effect, but had no effect on the study overall (Fig. 7A–D).

### Prediction of related drugs and miRNAs

**Fig. 8:**
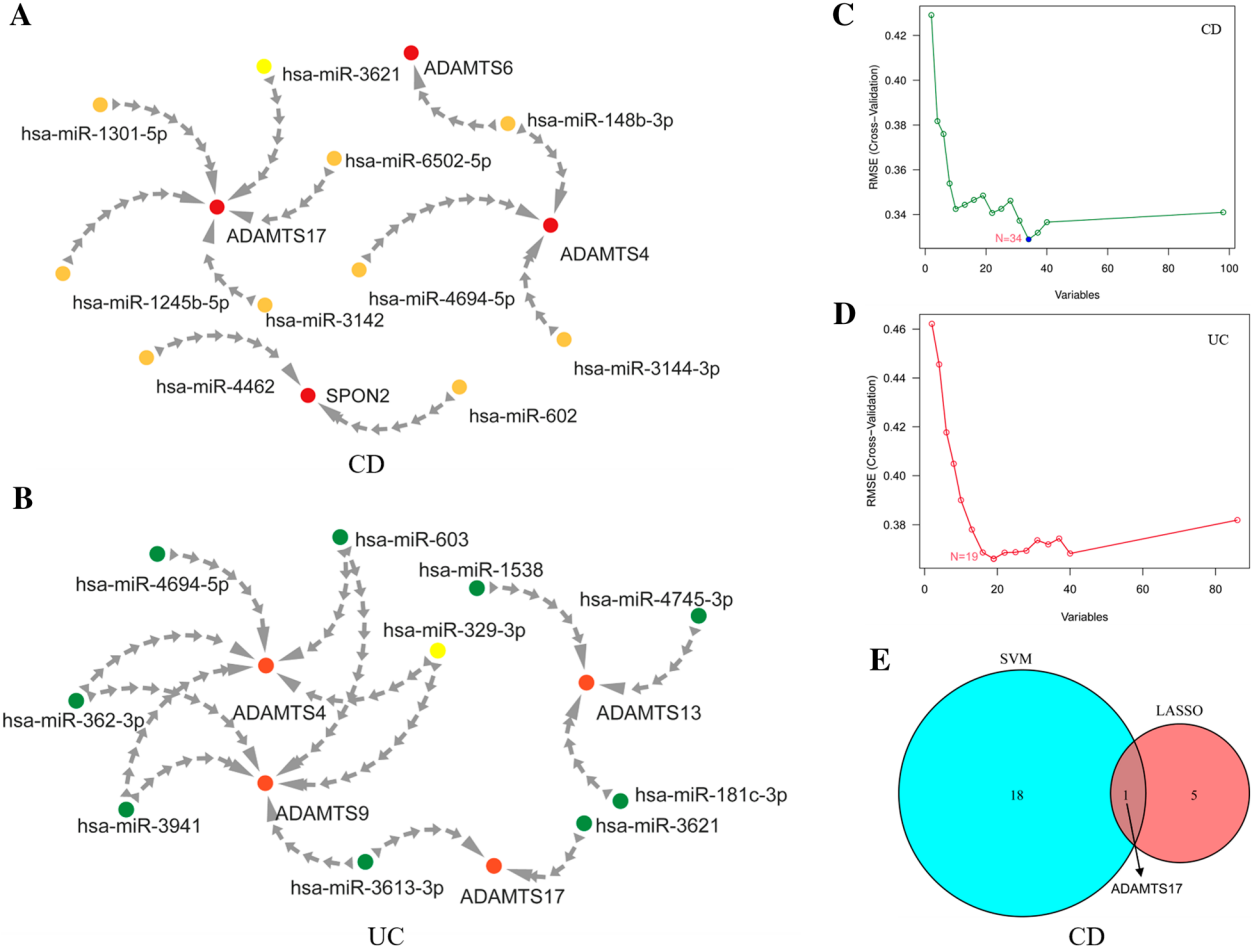
Prediction of relevant miRNAs and machine learning screening. **A**, **B** In the CD and UC groups, the BM hub gene sets were predicted for miRNA, and the correlation was visualized. **C**, **D** Machine learning was performed on the differential expression BM gene sets, respectively, and the central genes were screened by the model. In the CD group, the Lasso regression model and the genes screened by the machine learning model were subjected to Venn diagram intersection. **E** Venn plot intersection of BM-related hub genes screened by Lasso regression model and machine learning in CD group

We used the immune infiltration-related hub genes to predict related drugs and miRNAs through the online Enrichr database. It is a comprehensive online tool for gene enrichment analysis, containing a large library of genome annotations available for analysis and download, such as Transcription, Pathways, Ontologies, Diseases/Drugs and Cell types [28]. We entered the six important genes screened for drug correlation prediction, calculated the corresponding *p* values (*p* < 0.05), and performed reverse order to select the top 10 hub drugs for presentation (Table 2). Overlap represents how many genes are predicted to point to this drug, and higher values suggest better prediction for the disease. In the CD and UC groups, we obtained the most sensitive drugs were retinoic acid and arbutin, respectively. miRNA prediction data (*p* < 0.05) were also extracted, the genes associated with them were connected, and their regulatory forms were visualized by Cytoscape software. The red color indicated the names of hub BM genes (Fig. 8A, B). It will provide guidance and reference significance for disease treatment and in-depth research.

**Table 2:** Predicting related drugs for hub BM genes

CD group			UC group		
Drug	Overlap	*P*	Drug	Overlap	*p*
Retinoic acid	5/6	0.0022	Arbutin	2/6	0.0029
CGS-27023A	1/6	0.0039	CGS-27023A	1/6	0.0039
Trichostatin A	4/6	0.0114	AFLATOXIN B1	4/6	0.0065
VANADIUM	1/6	0.0439	Pivampicillin	1/6	0.0069
VALPROIC ACID	5/6	0.0486	Uridine Triphosphate	1/6	0.0102
–	–	–	Clonidine	1/6	0.0105
–	–	–	CAPECITABINE	1/6	0.0149
–	–	–	Clopidogrel Bisulfate	1/6	0.0199
–	–	–	Imidurea	1/6	0.0220
–	–	–	Methylprednisolone	1/6	0.0223

### Machine learning finds hub genes

We screen for hub genes through computer learning methods. In the CD group, a total of 18 meaningful hub genes were screened out and intersected with the predicted hub genes to obtain the central hub gene ADAMTS17 (Fig. 8C, E). The Venn diagram is used to represent overlapping regions between multiple data. In the UC group, a total of 34 significant hub genes were screened out and intersected by the Venn diagram to obtain the central hub genes ADAMTS17 and ADAMTS9. In the CD/UC group, a total of 40 significant hub genes were screened out and intersected by the Venn diagram to obtain the central hub genes POSTN, SPARC, and ADAMTS2 (Fig. 8D and Additional file 1: Fig. S5A, B).

## Discussion

In recent years, with the continuous research on IBD, the expected therapeutic effect has still not been achieved. The course of IBD is usually prolonged and even requires lifelong treatment. Its pathogenic mechanism is complex [29–31] and is closely related to the inflammatory immune response of the intestine [32]. Therefore, the in-depth study of its immune regulation mechanism has always been a clinical research hotspot. The pathological manifestations of IBD are mainly in the congestion and erosion of the intestinal tissue mucosa, the structure of the matrix is damaged, accompanied by the infiltration of a large number of inflammatory cells [33, 34]. BM is an extracellular matrix structure, which is formed by the combination of collagen and adhesions protein to form a tissue structure, which is closely related to the formation of the intestinal mucosal structure. Recently, Jayadev et al. [10] successfully identified the BM gene set by network analysis and screening of elegans and zebrafish, which finally clearly defined 222 human BM set and affirmed the important impact of BM genes on human health. It is composed of 160 basement members matrix and 62 cell surface interactors, which are the scaffolding structure of the tissue and play an important role as a protective barrier. However, there are fewer studies on BM genes on diseases, which the role of BM genes in IBD diseases has not been carried out, thus BM genes are important for IBD studies. We extracted the BM gene set for the study to discover the key pathogenic genes of IBD. A further prediction of related drugs and miRNAs has important clinical application and guiding significance for basic experiments.

We are interested in a multi-faceted exploration of IBD through BM genes. In both CD and UC, we were surprised to find that these two disease subtypes collectively focus on the ADAMTS family [35, 36]. At present, the ADAMTS family has been studied to be associated with the occurrence of various diseases and has a hub role in tissue development and homeostasis. Its functions are widely determined by their interactions with the extracellular matrix and proteins in the extracellular matrix [37]. It is also suggested that its functional form of action may be closely related to the histopathological destruction and immune cell infiltration of IBD. In CD, we further screened out the central hub gene ADAMTS17 by machine learning. It is related to fibrillin biology [38–41], and its downregulation is associated with dysplasia, and ECM substrates are functionally linked, consistent with our analysis that low expression promotes the development of CD [42]. It has also been found that there is a significant negative correlation with immune functions such as T lymphoid helper cells, paracrine, and type I interferon responses, which may be involved in the immune response of CD. In UC, central hub genes ADAMTS17 and ADAMTS9 were identified. Among them, ADAMTS9 belongs to the hub enzyme of proteoglycan degradation [43–45], and its harmful role in osteoarthritis, rheumatoid arthritis, and intervertebral disc degeneration have been widely described [46]. Possibly related to disrupting the function of the extracellular structural matrix, consistent with our analysis. In the immune response, it is significantly related to the promotion of B cells, CD8 + T cells, and DCs, the promotion of inflammatory responses, and the inhibition of NK cells and other immune cells. Chen et al. suggested that there is a strong interaction force between BM and immune cells, while regulating immune cell levels [11, 47, 48]. BM genes are also involved in the regulation of immune cells including mast cells and may be associated with the activation of loaded inflammatory networks including mast cells when inflammatory factors bind to the BM [49–51].

In addition, we are also interesting to find that there are also significant differences between CD and UC [52]. The clinical manifestations of CD and UC are different, but some patients are difficult to identify, especially in the early stage of the disease. Therefore, it is of great clinical significance to find new biomarkers for identification. We found that the BM-related differential expression genes of the two subtypes were still enriched in the cell–matrix organization and structural organization, which indicated that the degree of structural changes in the stromal organization was different between them [53]. The analysis showed that the hub genes of the difference center were SPARC, POSTN, and ADAMTS2. The gene expression levels of these three genes in UC were higher than those in CD, and the AUC of the SPARC gene was 0.71, indicating a more accurate prediction effect. It is mainly involved in the regulation of cell adhesion, proliferation, migration, and tissue remodeling, and is related to the expression of fibroblasts [54, 55]. This may suggest that the differences in the pathological and immune-inflammatory responses developed between the two are closely related.

Our model has a good prediction of the pathogenic factors of CD and UC, and also explains the differences between the two to a certain extent, which can guide clinical treatment and basic research plans. As our findings were derived from bioinformatic analysis, there are deficiencies in the authenticity and credibility of the findings without experimental evidence of protein, and further experimental studies are required to confirm these results.

## Conclusion

In conclusion, we constructed and validated a nomogram model of CD and UC composed of BM genes through a series of multiple group bioinformatics comparative analyses. In CD, the central key gene is ADAMTS17; in UC, the central key gene is ADAMTS17 and ADAMTS9, which are closely related to the progression of disease, hoping to provide a new direction for the diagnosis and treatment of IBD. Additionally interesting, we found that the important differential significance of SPARC, POSTN and ADAMTS2 between CD and UC, which was able to clarify the similarities and differences between CD and UC, provides new insights into the identification of IBD subtypes, and has important research significance.

## Supplementary Information

**Additional file 1.** Additional tables S1-S7.

**Additional file 2.** Additional figures S1-S5.

## Data Availability

The data used and analyzed in this study can be obtained from the corresponding author on reasonable request.

## Abbreviations

IBD: Inflammatory bowel disease
CD: Crohn's disease
UC: Ulcerative colitis
BM: Basement membrane
ROC: Receiver operating characteristic curve
AUC: Area under curve
GEO: Gene expression omnibus
DEGs: Differentially expressed genes
PPI: Protein–protein interaction
GO: Gene ontology
KEGG: The Kyoto Encyclopedia of Genes and Genomes
GSEA: Gene set enrichment analysis
ssGSEA: Single sample gene set enrichment analysis
SVM-RFE: Support vector machines-recursive feature elimination
