# Supplementary material for: Screening of hub inflammatory bowel disease biomarkers and identification of immune-related functions based on basement membrane genes
Source: Eur J Med Res. 2023 Jul 22;28:247. doi: 10.1186/s40001-023-01193-5 (PMC10362583; doi:10.1186/s40001-023-01193-5)
Supplement: Supplementary file 2 — Additional file 2. Additional figures S1-S5. [file 40001_2023_1193_MOESM2_ESM.doc]

**Screening of hub inflammatory bowel disease biomarkers and identification of immune-related functions based on basement membrane geness**

Penghang Lin^1*^, Jin Hua^1*^, Zuhong Teng^1*^, Chunlin Lin^1,2^, Songyi Liu^1,2^, Ruofan He^1,2^, Hui Chen^1,2^, Hengxin Yao^1,2^, Jianxin Ye^1#^, Guangwei Zhu1#,

1. Department of Gastrointestinal Surgery 2 Section, Institute of Abdominal Surgery, Key Laboratory of accurate diagnosis and treatment of cancer, The First Affiliated Hospital of Fujian Medical University, National Regional Medical Center, Binhai Campus of the First Affiliated Hospital, Fujian Medical University, Fuzhou 350005, China;2. Key Laboratory of Ministry of Education for Gastrointestinal Cancer, Fujian Medical University, Fuzhou 350000, China.

#Corresponding author: Jian-Xin Ye, Department of Gastrointestinal Surgery 2 Section, Institute of Abdominal Surgery, Key Laboratory of Accurate Diagnosis and Treatment of Cancer, The First Hospital Affiliated to Fujian Medical University, 20th, Chazhong Road, Fuzhou, Fujian 350005, China. Tel: +86-138-0955-3280; E-mail: yejianxinfuyi@126.com

Guangwei Zhu, Department of Gastrointestinal Surgery 2 Section, Institute of Abdominal Surgery, Key Laboratory of Accurate Diagnosis and Treatment of Cancer, The First Hospital Affiliated to Fujian Medical University, 20th, Chazhong Road, Fuzhou, Fujian 350005, China. Tel: +86-180-6047-3703; E-mail: zgwzsy@126.com

*These authors contributed equally to this study.

**Abstract**

**Background:** Inflammatory bowel disease (IBD), including Crohn's disease (CD) and ulcerative colitis (UC), is a chronic, inflammatory, and autoimmune disease, but its specific etiology and pathogenesis are still unclear. This study aimed to better discover the causative basement membrane (BM) genes of their subtypes and their associations.

**Methods:** The differential expression of BM genes between CD and UC were analyzed and validated by downloading relevant datasets from the GEO database. We divided the samples into 3 groups for comparative analysis. Construction of PPI networks, enrichment of differential gene functions, screening of Lasso regression models, validation of ROC curves, nomogram for disease prediction and other analytical methods were used. The immune cell infiltration was further explored by ssGSEA analysis, the immune correlates of hub BM genes were found, and finally, the hub central genes were screened by machine learning.

**Results:** We obtained 6 candidate hub BM-related genes related to cellular immune infiltration in the CD and UC groups respectively, and further screened the central hub genes ADAMTS17 and ADAMTS9 through machine learning. In all ROC curve models, AUC>0.5, indicating that this characteristic gene has a more accurate predictive effect on IBD. We also found that the pathogenicity-related BM genes of the CD and UC groups were mainly concentrated in the ADAMTS family (ADAMTS17 and ADAMTS9). There are some differences between the two subtypes, and the central hub BM-related genes are SPARC, POSTN, and ADAMTS2.

**Conclusions:** In the current study, for the first time, we provided a nomogram model of CD and UC composed of BM genes, identified central hub genes, and clarified the similarities and differences between CD and UC. This will have potential value for preclinical, clinical, and translational guidance and differential research in IBD.

**Keywords:** basement membrane, IBD, biomarkers, CD, UC, SVM-RFE

**Supplementary Figure Legends**


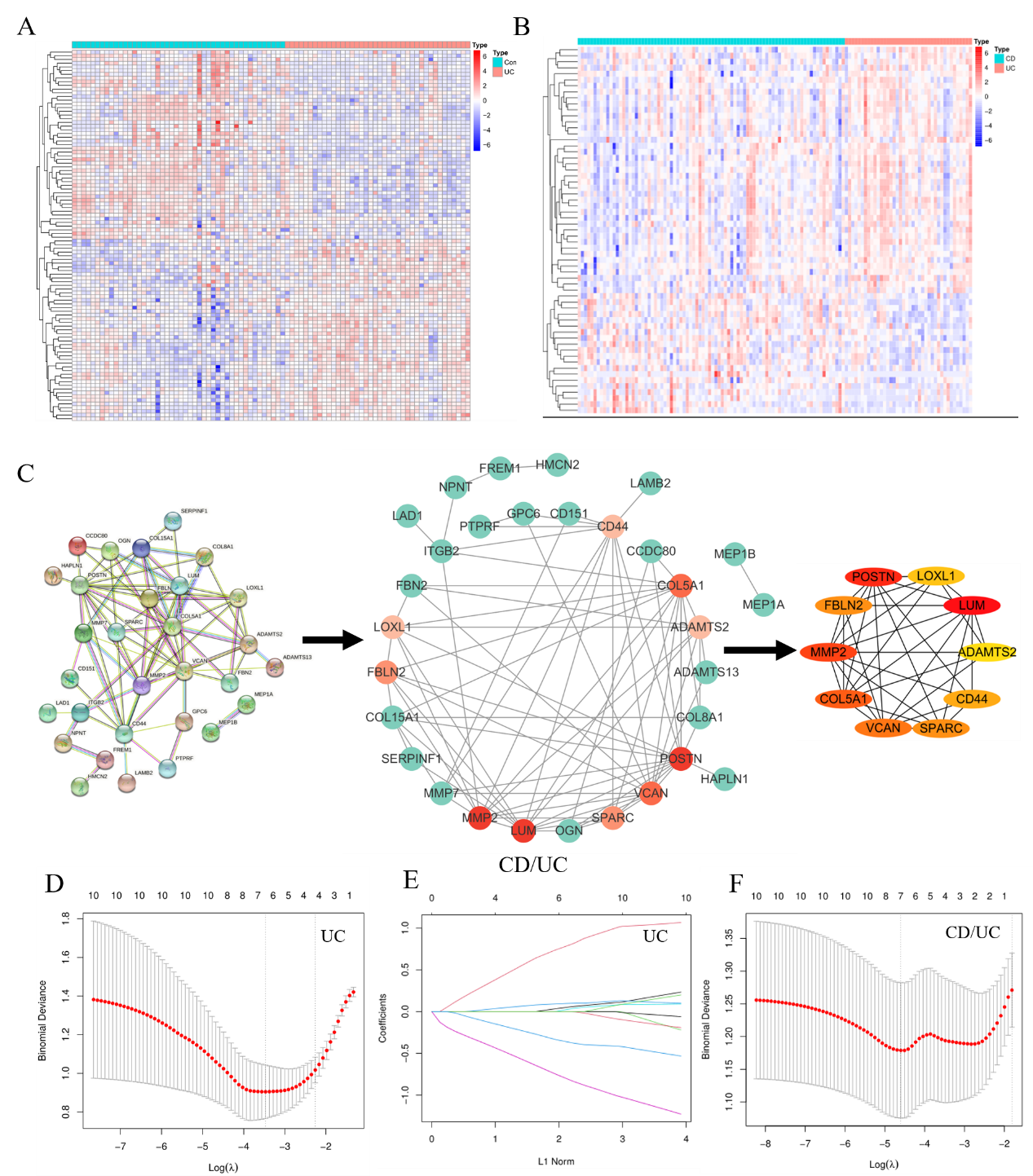


**Fig. S1** Screening of hub BM-related DEGs. (A, B) Heatmap of differential expression of BM-related genes in UC group and CD/UC group. (C) PPI network construction of BM-related differential genes in CD/UC group. (D) LASSO coefficient profile of 10-fold cross-validation hub genes in the UC group. (E) Partial likelihood bias of log-change plotted by LASSO regression in 10-fold cross-validation. A dashed vertical line is drawn at the optimal value with the minimum criterion (lambda. min) and 1 standard error of the minimum criterion (1-se criterion). (F) LASSO coefficient profile of 10-fold cross-validation hub genes in CD/UC group.


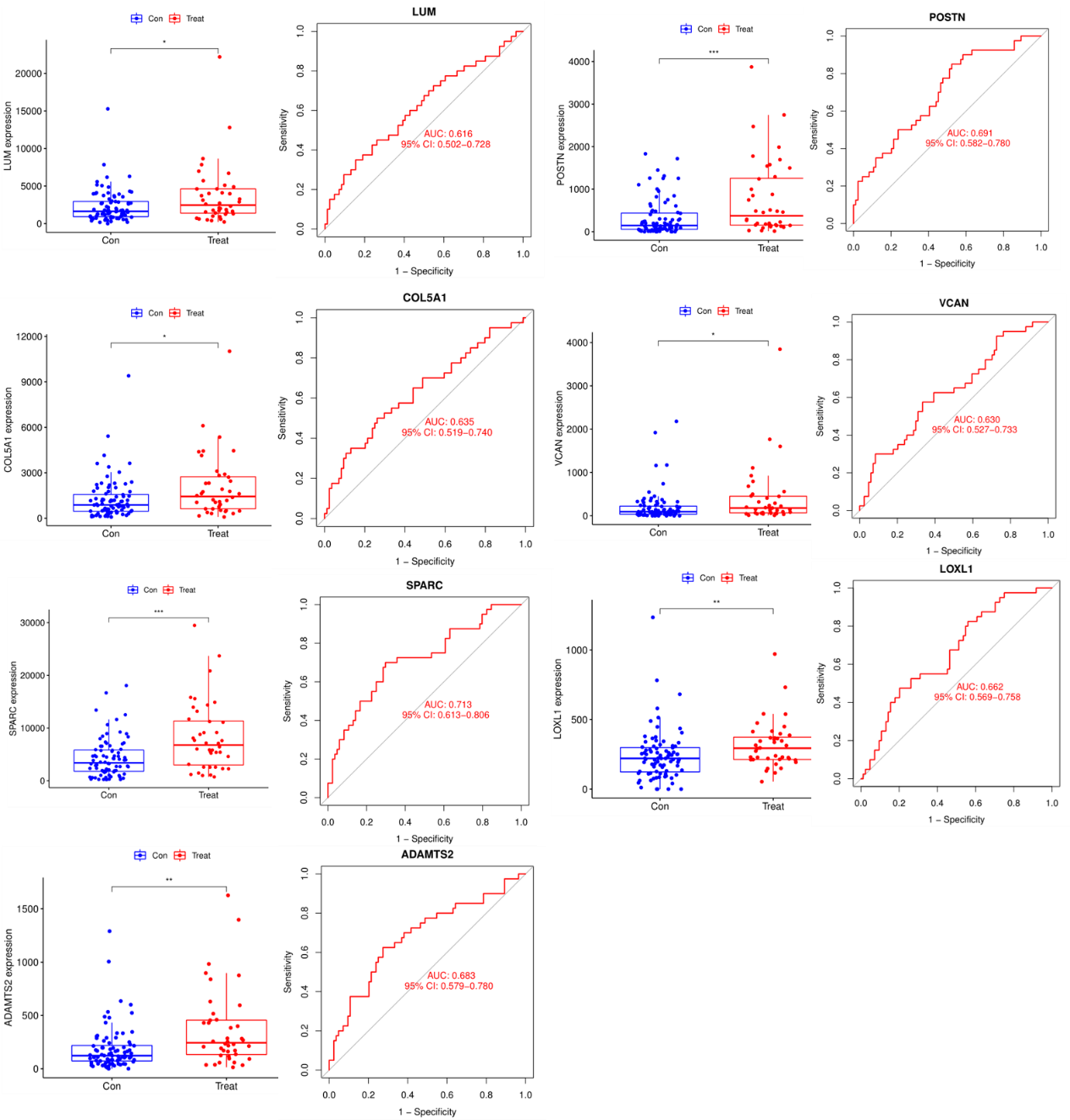


**Fig. S2** In the CD/UC group, the hub BM gene boxplots and ROC curve models were drawn. (*p < 0.05, *p < 0.05, *p < 0.05, with an unpaired Student’s t -test)


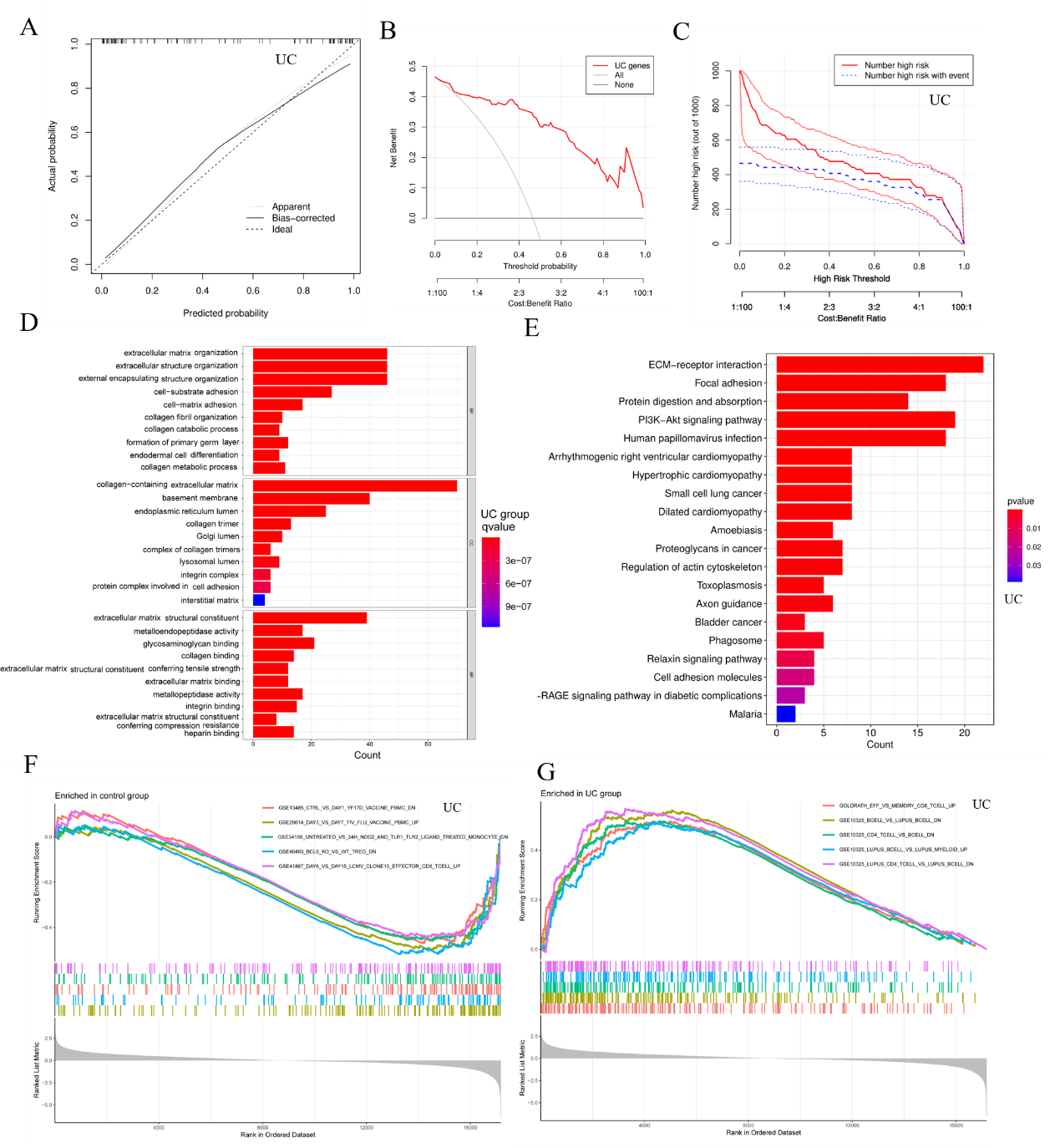


**Fig. S3** Functional analysis of DEGs. (A-C) Prediction curves, decision curves, and clinical impact curves of the nomogram model in the UC group. (D, E) Bar graphs of GO and KEGG enrichment analysis of differential BM-related genes in the UC group. (F, G) In the UC group analysis, GSEA analysis was performed with the immune marker gene set as a reference, and the top five enriched immune gene sets were listed respectively.


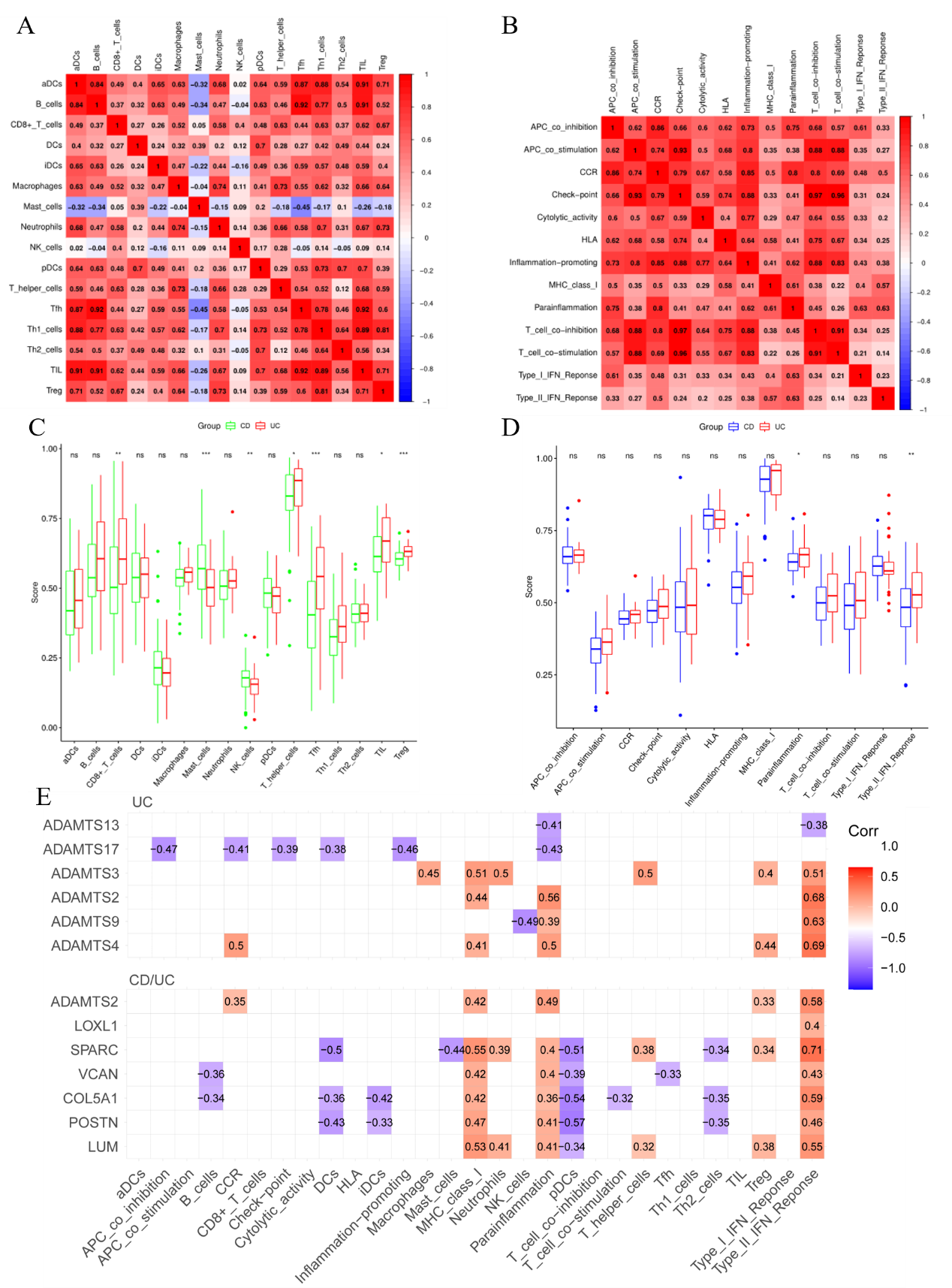


**Fig. S4** Immune cell and functional correlation analysis. (A, B) Correlation of immune cells and immune function in the UC group. (C, D) Expression of immune cells and immune function in CD/UC group. (E) Relationship of hub BM-related genes with immune cell infiltration in UC and CD/UC groups. (*p < 0.05, *p < 0.05, *p < 0.05, with an unpaired Student’s t -test)


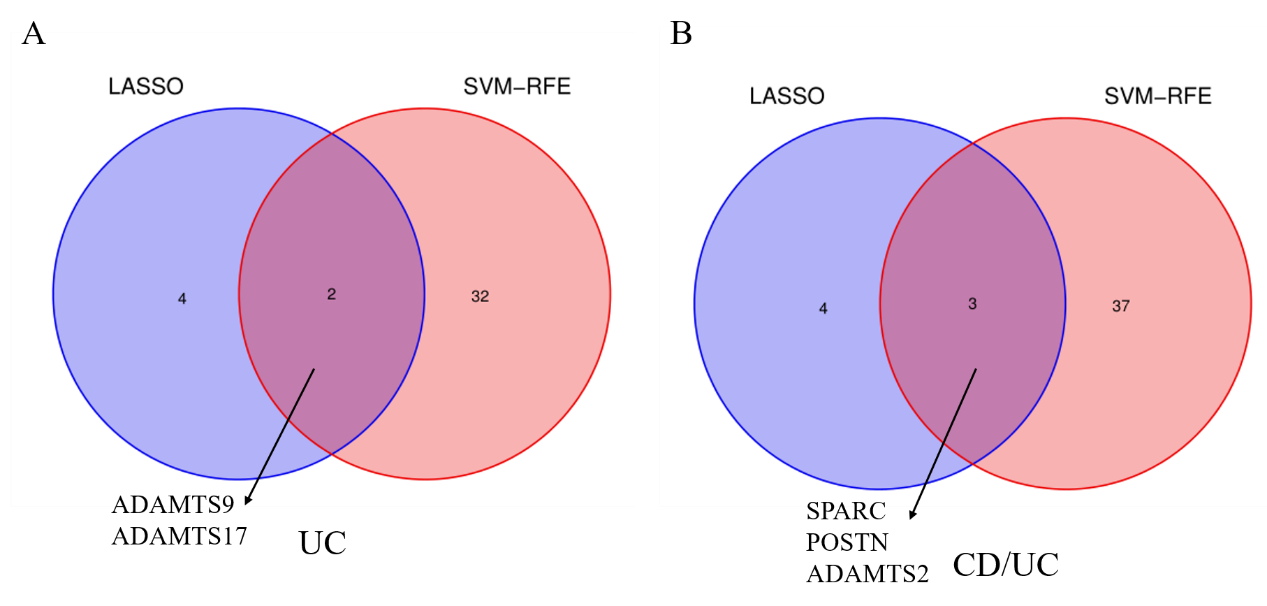


**Fig. S5** Screening of hub BM-related genes in the center. (A, B) Venn plot intersection of BM-related hub genes screened by Lasso regression model and machine learning in UC and CD/UC groups.
